# Effects of Treatment with Glucagon-like Peptide-1 Receptor Analogues on the Diabetic Foot

**DOI:** 10.3390/biomedicines14020406

**Published:** 2026-02-10

**Authors:** Mercedes Ortiz Romero, David Rodríguez de Vera Gómez, Pablo Rodríguez de Vera Gómez, Luis María Gordillo Fernández

**Affiliations:** 1 Podiatry Faculty, University of Seville, 41009 Seville, Spain; mortiz17@us.es (M.O.R.); lgordillo@us.es (L.M.G.F.); 2 Endocrinology and Nutrition Department, Virgen Macarena University Hospital, 41009 Sevilla, Spain; pablordevera@gmail.com

**Keywords:** diabetic foot disease, diabetic peripheral neuropathy, GLP-1 receptor agonists, pleiotropic effects, lower-limb complications, hospitalisations, type 2 diabetes mellitus

## Abstract

**Background/Objectives**: Diabetic foot disease is one of the most severe and disabling complications of type 2 diabetes mellitus, resulting from the interaction between peripheral neuropathy, peripheral arterial disease, and infection. It is associated with a high risk of ulceration, lower-limb amputation, hospitalisation, and mortality, and is currently recognised as a marker of advanced systemic vascular disease. Although glucagon-like peptide-1 receptor agonists have demonstrated robust cardiometabolic benefits, their potential impact on diabetic foot disease outcomes remains insufficiently explored. **Methods**: This narrative review critically synthesises clinical, experimental, and translational evidence evaluating the association between glucagon-like peptide-1 receptor agonist therapy and diabetic foot disease-related outcomes. A comprehensive literature search was conducted in PubMed and related databases, focusing on studies published over the last decade that assessed diabetic peripheral neuropathy, foot ulceration, amputations, hospitalisations, and mechanistic pathways potentially linking glucagon-like peptide-1 receptor agonists to diabetic foot pathophysiology. **Results**: Available observational studies and population-based analyses suggest that glucagon-like peptide-1 receptor agonist treatment is associated with a reduced incidence of diabetic foot ulcers, lower-limb amputations, and related hospitalisations. Experimental and translational data provide biological plausibility for these findings, demonstrating neuroprotective effects, attenuation of neuroinflammation, and improvement of endothelial function and microvascular perfusion, as well as modulation of inflammatory and reparative pathways involved in wound healing. These pleiotropic actions extend beyond glycaemic control and may influence the natural history of diabetic foot disease. **Conclusions**: Glucagon-like peptide-1 receptor agonists emerge as promising therapeutic agents with potential benefits in the prevention and progression of diabetic foot disease. Their integrated neurovascular and immunometabolic effects may contribute to improved clinical outcomes and a reduced healthcare burden. Prospective studies and dedicated clinical trials are warranted to confirm these associations and to define the role of glucagon-like peptide-1 receptor agonists in the multidisciplinary management of diabetic foot disease.

## 1. Introduction

Diabetic foot disease (DFD) represents one of the most severe and complex complications of diabetes mellitus (DM), resulting from the interaction between peripheral neuropathy, peripheral arterial disease, and infection, leading to ulceration, tissue destruction and, in advanced cases, lower-limb amputation [1,2]. It is estimated that 15–25% of individuals with diabetes will develop a foot ulcer during their lifetime, and that more than 80% of non-traumatic amputations are preceded by an active ulcer [3]. Despite advances in multidisciplinary management, the treatment of diabetic foot disease remains a major clinical challenge due to high recurrence rates, difficulties in achieving complete wound healing in the presence of neuropathy and ischemia, and the substantial burden of hospitalisations, mortality and associated healthcare costs [4,5]. In this context, diabetic foot disease is now recognised not only as a localised complication, but also as a marker of systemic vascular disease and poor overall prognosis in patients with type 2 diabetes mellitus (T2DM) [6].

Glucagon-like peptide-1 receptor agonists (GLP-1RAs) have represented a paradigm shift in the treatment of T2DM over the past decade. Initially developed for their incretin effect on glucose-dependent insulin secretion, these agents have demonstrated clinical benefits that extend well beyond glycaemic control, including sustained weight loss, reduction in major adverse cardiovascular events and renal protection in high-risk populations [7,8,9]. In addition, recent studies have highlighted their pleiotropic effects on inflammation, lipid metabolism, endothelial function, and the nervous system, which has generated increasing interest in their potential modulatory role in chronic complications of DM that are traditionally difficult to manage, such as peripheral neuropathy and diabetic foot disease [10,11,12].

However, despite robust evidence supporting the cardiovascular and metabolic benefits of GLP-1RAs, their specific impact on diabetic foot disease remains insufficiently explored. Most pivotal clinical trials did not include outcomes focused on the lower extremities, peripheral neuropathy or hospitalisations related to diabetic foot ulcers [13]. The available evidence derives mainly from observational studies, population-based database analyses and translational research, which suggest a potential reduction in the risk of foot ulcers, amputations and limb-related events in patients treated with GLP-1RAs, but without a systematic synthesis specifically focused on the pathophysiology and clinical course of diabetic foot disease [12,14,15]. Furthermore, the neurovascular mechanisms that could account for these potential benefits have not yet been clearly integrated within the context of diabetic foot disease.

Within this framework, the aim of the present review is to critically analyse the available evidence regarding the association between treatment with GLP-1 receptor agonists and diabetic foot disease, with particular emphasis on peripheral neuropathy, the underlying pathophysiological mechanisms and clinically relevant outcomes such as ulcer development, amputations, and hospitalisations. By integrating clinical, experimental, and translational data, this review seeks to provide an updated perspective on the potential role of GLP-1RAs as modulators of the natural history of diabetic foot disease and to identify key areas for future research within the field of novel therapeutic strategies for diabetic neuropathy.

## 2. Materials and Methods

A comprehensive literature search was performed using PubMed/MEDLINE and Google Scholar as the primary electronic databases. The search strategy combined Medical Subject Headings (MeSH) terms and free-text keywords related to T2DM, diabetic foot, diabetic foot ulcer, peripheral neuropathy, lower-limb amputation, hospitalisation, incretin-based therapy, and GLP-1 RAs.

The search was restricted to articles published between January 2008 and March 2025, in order to capture both foundational mechanistic studies and the most recent clinical and observational evidence, particularly large population-based cohorts and systematic reviews. Only articles published in English were considered. Eligible studies included randomised controlled trials, observational cohort and case–control studies, database analyses, systematic reviews, and meta-analyses. Preclinical experimental studies were also included when they provided relevant mechanistic insights into vascular, neural, inflammatory, or reparative pathways potentially involved in diabetic foot pathology.

Titles and abstracts were initially screened to assess relevance, followed by the full-text evaluation of selected manuscripts. Studies focusing exclusively on glycaemic outcomes without reference to neuropathy, foot complications, vascular endpoints, or hospitalisation were excluded. Additional relevant publications were identified through manual screening of reference lists from key articles. Given the heterogeneity of study designs, populations, and outcome definitions, a formal meta-analysis was not undertaken. Instead, findings were synthesised qualitatively, with particular attention to consistency of results across different study types and to the biological plausibility of the proposed mechanisms.

The review adhered to established principles for narrative reviews, aiming to integrate clinical outcomes with mechanistic data to provide a coherent interpretation of how GLP-1 RAs may influence the natural history of diabetic foot disease and its associated burden on hospital care.

Overall, the literature search retrieved 1284 records. After the removal of duplicates (n = 468) and exclusion of studies that did not meet the eligibility criteria, a total of 39 articles were included for qualitative synthesis. Of these, 4 were clinical trials, 4 were meta-analyses or systematic reviews, and 8 were observational studies; the remaining articles consisted of clinical guidelines, narrative reviews, and preclinical studies used to contextualise and support the mechanistic framework discussed.

## 3. Results

### 3.1. Diabetic Foot Disease: Peripheral Neuropathy

Peripheral diabetic neuropathy constitutes the central pathogenic factor of DFD [16]. Sensory involvement leads to the loss of pain, temperature, and pressure perception, rendering the foot vulnerable to minor, often unnoticed trauma. Motor neuropathy results in muscle atrophy, digital deformities (such as hammer toes or claw toes), bony prominences, and abnormal redistribution of plantar pressures, which favours the development of hyperkeratosis and subsequent pressure-related ulceration at sites of maximal load [17]. In addition, autonomic neuropathy reduces sweating and leads to atrophic, dry, and fissured skin, which represents a frequent portal of entry for bacterial infection. Furthermore, increased blood flow through arteriovenous shunts contributes to a warm, oedematous foot with enhanced bone resorption, predisposing to the development of Charcot neuroarthropathy in advanced cases [2,17].

Lesions associated with diabetic foot disease are frequently neuropathic or neuro-ischaemic, reflecting the coexistence of some degree of peripheral arterial disease (PAD) in the majority of patients with diabetes [17]. When present, ischemia compromises wound healing and facilitates progression to gangrene or limb amputation. The combination of neuropathy, foot deformity, and repetitive microtrauma during gait explains why many ulcers develop in the absence of an identifiable external injury [16,18,19].

Patients with neuropathy and/or a history of lower-limb amputation exhibit a higher risk of cardiovascular disease, chronic kidney disease, and all-cause mortality [17,20]. The role of peripheral neuropathy in the pathophysiology of diabetic foot disease is summarised in Table 1.

Diabetic peripheral neuropathy constitutes the core pathophysiological mechanism of diabetic foot disease, as it disrupts the sensory, motor, and autonomic integrity of the foot, thereby facilitating the development of lesions that, in the presence of additional risk factors, may progress to lower-limb amputation.

### 3.2. Impact on Hospital Admissions

Diabetic foot disease represents one of the leading causes of hospitalisation among patients with T2DM, owing to the high incidence of deep infections, ulceration, and progression to lower-limb amputation [2,5]. Hospital admissions due to diabetic foot ulcers or infections constitute a marker of clinical severity and are consistently associated with a substantial increase in mortality, disability, and healthcare costs [5,21,22].

The most robust population-based data derive from large hospital databases. In Spain, an analysis of more than 15,000 hospitalisations for diabetic foot infections showed that these admissions account for approximately 0.4% of all internal medicine hospital stays, yet they concentrate an in-hospital mortality rate close to 16% and an amputation rate of 8.25% per episode [5].

Other studies focusing on the first hospitalised episode of diabetic foot ulceration have shown that the initial admission represents a critical turning point, as the combined incidence of death and amputation in the subsequent months is remarkably high [21].

At a global level, epidemiological evidence allows for the true magnitude of this condition to come into focus. A meta-analysis of observational studies encompassing multiple regions worldwide estimated that the 5-year mortality rate among patients with active diabetic foot ulceration approaches 50%, comparable to or exceeding that of several solid malignancies [2,23]. Mortality is even higher in cases complicated by deep infection, critical limb ischemia or previous amputation [6,21,22].

Diabetic foot infection, one of the principal drivers of hospitalisation, frequently requires broad-spectrum empirical antibiotic therapy, multidisciplinary management and, in a substantial proportion of cases, surgical intervention, or extensive debridement [5]. Recent hospital-based studies indicate that the complexity of infectious management increases length of stay, episode-related costs, and the risk of short-term readmission [24].

Alongside classical epidemiological studies, recent years have seen the emergence of analyses evaluating how different antidiabetic treatments may influence the occurrence of severe lower-extremity events, including hospitalisations [25]. In this context, growing attention has focused on GLP-1 RAs and their potential impact on the clinical course of diabetic foot disease [14,15].

The study by Werkman et al. [14], one of the most robust observational investigations available, used the CPRD Aurum database to compare GLP-1 RAs with other glucose-lowering agents in patients with T2DM. The results showed that incretin use was associated with a significant reduction in the risk of diabetic foot ulceration, amputations, and hospitalisations related to this condition, compared with other antidiabetic treatments [14].

A similar trend was observed in an analysis of the TriNetX network, in which patients with active diabetic foot ulcers treated with semaglutide experienced a significant reduction in complications, such as non-healing wounds, recurrent infections, and both minor and major amputations. Although hospitalisations were not directly assessed, the overall reduction in severe foot-related events serves as an indirect indicator of a lower need for inpatient care [26].

Likewise, a large comparative population-based study, which matched more than 180,000 patients treated with GLP-1 RAs to an equivalent number treated with SGLT2 inhibitors, demonstrated that GLP-1 RAs were associated with a lower incidence of major and minor amputations, new episodes of diabetic foot ulceration, and mortality [15,27]. Although hospitalisation was not the primary endpoint, these lower-extremity outcomes represent the most frequent causes of admission in this population, and their reduction has direct clinical implications for the hospital burden of diabetic foot disease.

Consistent findings have also been reported in very high-risk populations, such as patients with peripheral arterial disease or active foot ulcers. In the study by Caruso et al., semaglutide use was associated with a 23% reduction in major adverse limb events—including revascularisation, critical limb ischemia, and lesion progression—and a 50% reduction in amputations. These findings suggest that GLP-1 RAs may play a meaningful role in modifying prognosis and, potentially, in reducing hospitalisations due to diabetic foot complications [28].

Finally, a meta-analysis involving more than two million patients compared the risk of amputation among users of SGLT2 inhibitors, DPP-4 inhibitors, and GLP-1 RAs, showing that the latter are not only safe but may confer a more favourable profile with respect to lower-extremity events [25]. Given that amputations constitute the leading cause of prolonged hospitalisation in diabetic foot disease, the reduction in this outcome further supports the hypothesis that GLP-1 RAs may be associated with a lower hospital burden. The most relevant primary clinical and observational studies evaluating the association between GLP-1 RA therapy and diabetic foot-related outcomes are summarised in Table 2.

### 3.3. Biological Mechanisms Underlying the Effects of GLP-1 Receptor Agonists

The clinical outcomes described above, such as a lower incidence of diabetic foot ulcers, amputations, and hospitalisations in patients treated with GLP-1 RAs, are consistent with a set of pleiotropic mechanisms acting at metabolic, vascular, immunological, and neurological levels, beyond simple glycaemic control. These mechanisms may explain the observed benefits on diabetic foot disease from both vascular and neurological perspectives through several complementary hypotheses.

First, it is well established that GLP-1 RAs exert direct neuroprotective effects on the peripheral nervous system. Recent clinical studies in patients with T2DM and peripheral neuropathy have shown that prolonged treatment with GLP-1 RAs is associated with improvements in tibial nerve morphology, including reductions in nerve cross-sectional area and fascicular oedema, increased sensory nerve action potential amplitudes, and improved clinical neuropathy scores, suggesting structural and functional repair of both large and small nerve fibres [24]. In animal models of diabetic neuropathic pain, the administration of semaglutide significantly reduces mechanical allodynia and thermal hyperalgesia, with an analgesic effect accompanied by decreased levels of pro-inflammatory cytokines (TNF-α, IL-1β and IL-6) in the spinal cord and marked inhibition of microglial and astrocytic activation, even in the presence of modest changes in glycaemia, supporting a direct central effect on neuroinflammation [22]. These findings are consistent with earlier studies demonstrating the abundant expression of GLP-1 receptors in dorsal horn microglia and showing that their activation attenuates pain hypersensitivity through β-endorphin release and the modulation of endogenous opioid pathways, establishing a GLP-1R/β-endorphin inhibitory pathway specific to chronic pain states [29].

In parallel, there is robust mechanistic evidence linking GLP-1 signalling to peripheral neuronal protection and neurogenesis. Integrative reviews indicate that GLP-1 and its agonists reduce oxidative stress and neuronal apoptosis, modulate survival pathways, downregulate NF-κB activation, and promote neurogenesis in multiple regions of the central and peripheral nervous systems, including structures involved in nociception and motor control [30,31]. When extrapolated to diabetic foot disease, these effects could translate into a slower progression of peripheral neuropathy, preservation of protective sensation, reduced gait instability, and abnormal plantar pressure distribution, ultimately lowering the risk of repetitive microtrauma, ulceration, and the need for hospitalisation due to infection or acute complications.

Concurrently, GLP-1 RAs exert relevant effects on the endothelium and microcirculation, which are central to the ischaemic pathophysiology of the diabetic foot. Experimental studies and small clinical trials have shown that these agents improve cardiovascular risk parameters, including HbA1c, body weight, and blood pressure, and in some contexts are associated with trends towards improved coronary flow reserve or vascular function markers, although results regarding coronary microcirculation and peripheral endothelial function have been heterogeneous [10]. Nevertheless, mechanistic investigations demonstrate that GLP-1 receptor activation in endothelial and vascular smooth muscle cells reduces oxidative stress, attenuates the expression of adhesion molecules and pro-inflammatory cytokines, and increases nitric oxide bioavailability, thereby preserving endothelial homeostasis and limiting diabetes-related microvascular dysfunction. Although most data derive from coronary or systemic vascular beds, it is reasonable to hypothesise that similar effects at the level of distal foot microcirculation—arterioles, capillaries, and the vasa nervorum—could enhance oxygen and nutrient delivery to ischaemic tissue and peripheral nerves, improving tissue viability and wound-healing capacity [32].

Evidence related to wound healing provides a third pillar to support these hypotheses. Preclinical rodent models have shown that topical or systemic administration of GLP-1 RAs, such as liraglutide or exendin-4, accelerates wound closure in both normoglycaemic and diabetic animals. Reported mechanisms include increased capillary density and upregulation of VEGF-A and other angiogenic mediators, as well as modulation of macrophage polarisation from pro-inflammatory M1 phenotypes towards reparative M2 phenotypes across different phases of wound healing [32]. Furthermore, GLP-1 RAs promote keratinocyte migration through activation of the PI3K/Akt pathway, stimulate fibroblast accumulation and differentiation into myofibroblasts via ERK1/2 signalling, and enhance granulation tissue formation and re-epithelialisation, alongside the downregulation of NF-κB and TNF-α and the activation of AMPK and other cytoprotective pathways [11,32,33]. A recent review focusing specifically on diabetic foot ulcers highlights how these immunomodulatory, angiogenic, and tissue-repair effects may address several of the classical bottlenecks in diabetic foot ulcer healing, which is typically characterised by chronic sterile inflammation and ineffective angiogenesis [11].

From an integrative perspective, the convergence of these neural, vascular, and immunoregenerative effects provides a coherent pathophysiological framework to explain the reduction in clinical events observed in cohort studies and large database analyses, where GLP-1 RA use is associated with a lower risk of diabetic foot ulcer development, amputations, and diabetes-related foot hospitalisations. While part of this benefit may be mediated by improvements in glycaemic control, body weight, and cardiometabolic profile, experimental and translational evidence support an additional role for GLP-1 signalling in peripheral nerve protection, stabilisation of distal microcirculation, and enhancement of wound healing. This convergence of mechanisms reinforces the plausibility of considering GLP-1 RAs as a therapeutic strategy with a potential direct impact on the natural history of diabetic foot disease and supports the need for the formal evaluation of these agents in trials specifically designed with endpoints focused on peripheral neuropathy, foot ulcers, and foot-related hospitalisations.

The main preclinical and clinical studies supporting these mechanisms are summarised in Table 3 and Table 4.

## 4. Discussion

In the present review, we synthesise emerging clinical and translational evidence suggesting that glucagon-like peptide-1 receptor agonists (GLP-1RAs) may exert a favourable impact on diabetic foot pathology, particularly in relation to peripheral neuropathy, ulcer progression, and hospitalisation-related outcomes. Although GLP-1RAs were initially developed as glucose-lowering agents, accumulating data indicate that their effects extend beyond glycaemic control, encompassing vascular, neuroprotective, and immunomodulatory mechanisms that are highly relevant to the pathophysiology of diabetic foot disease.

**Figure 1:**
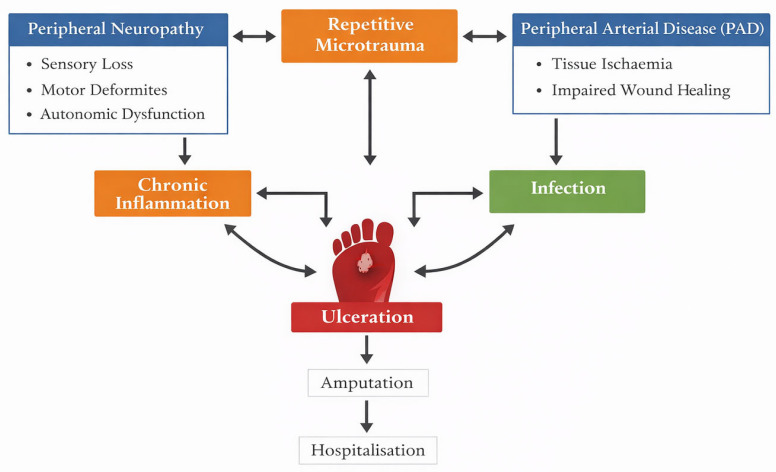
Pathophysiological mechanisms involved in diabetic foot disease.

The main mechanisms involved in the development and progression of diabetic foot disease are summarised in Figure 1.

### 4.1. Diabetic Foot Disease as a Systemic Condition

Diabetic foot disease should not be regarded as a purely local complication, but rather as a marker of advanced systemic metabolic and vascular deterioration. Several clinical features of diabetic foot disease—including persistent inflammation, neuropathic pain, recurrent infection, and functional impairment—contribute to an increased global cardiovascular risk. In this regard, amputations related to diabetic foot disease are considered true major cardiovascular events, as they represent the final stage of a process integrating systemic vascular damage, tissue ischemia, and advanced neuropathy [17,18]. Accordingly, both epidemiological analyses and recent observational studies and meta-analyses concur that hospitalisations related to diabetic foot disease should be regarded as major clinical events, comparable in impact to admissions for cardiovascular disease [2,6,23].

This adverse clinical profile is further amplified in the presence of chronic inflammation, infection, peripheral arterial disease (PAD), and suboptimal glycaemic control. In their analysis, Młynarska et al. highlighted how sustained hyperglycaemia, systemic inflammation, mitochondrial dysfunction, and oxidative stress act synergistically in both the progression of neuropathy and the acceleration of vascular damage, reinforcing the integrative concept of diabetic foot disease as a peripheral manifestation of systemic metabolic disease [20]. Consistently, hospitalisation for diabetic foot disease predicts significant subsequent clinical deterioration and increased mortality risk [21]. At a population level, epidemiological studies also reveal substantial disparities in hospitalisation, amputation, and mortality rates across different social, ethnic, and economic groups, further emphasising the disproportionate healthcare burden associated with this condition [22].

Beyond patient-related factors, the healthcare organisation plays a decisive role in clinical outcomes. Specialised multidisciplinary models of care have been shown to reduce amputation rates and hospital stays, thereby decreasing hospital burden and improving long-term prognosis [22]. These observations highlight that diabetic foot disease represents not only a clinical challenge but also a marker of healthcare system performance.

### 4.2. Pathophysiological Mechanisms of GLP-1 Receptor Agonists

GLP-1RAs exert a wide range of biological effects owing to the widespread expression of GLP-1 receptors in multiple organs, including the brain, heart, pancreas, gastrointestinal tract, skeletal muscle, and bone [34,35,36]. This broad tissue distribution underpins their pleiotropic nature, whereby a single molecular signalling pathway influences multiple phenotypic processes and enables modulation of metabolic, inflammatory, and cardiovascular pathways extending beyond glycaemic control. These integrated actions are directly relevant to the development and progression of chronic complications of diabetes mellitus, particularly diabetic neuropathy and diabetic foot disease, through effects on neural function, vascular homeostasis, and systemic inflammation [10,34,35,36].

Within the central nervous system, GLP-1RAs regulate appetite through hypothalamic pathways by stimulating anorexigenic signalling and inhibiting orexigenic circuits, leading to enhanced satiety, sustained appetite suppression, and reduced caloric intake. This contributes to clinically meaningful weight loss and a reduction in the systemic inflammatory burden associated with excess adipose tissue [34,35]. At the gastrointestinal level, GLP-1RAs delay gastric emptying via vagal-mediated mechanisms, attenuating postprandial glycaemic excursions and supporting improved metabolic control [34]. Although tachyphylaxis has been described, delayed gastric emptying appears to remain clinically relevant during long-term treatment.

In the pancreas, GLP-1RAs act as potent incretin hormones by enhancing glucose-dependent insulin secretion while suppressing inappropriate glucagon release. This dual action promotes stable glycaemic control, reduces glucotoxic stress, and indirectly attenuates pro-inflammatory and pro-oxidative processes involved in the progression of diabetic neuropathy [35,36]. Beyond these classical metabolic effects, GLP-1RAs exert systemic actions that are highly relevant to cardiovascular risk and microvascular disease. Clinical studies have demonstrated reductions in inflammatory markers and improvements in functional capacity in conditions such as heart failure, suggesting favourable interactions with vascular and metabolic pathways implicated in diabetic foot disease, including tissue ischemia, chronic inflammation, and impaired tissue repair [13].

From a neurological perspective, preclinical and clinical studies consistently show that GLP-1RAs reduce neuroinflammation, oxidative stress, and neuronal apoptosis while promoting structural and functional recovery of peripheral nerves [2,3,16,37]. Experimental models have demonstrated the attenuation of microglial activation, decreased pro-inflammatory cytokine expression, and improvements in nociceptive thresholds, effects that appear at least partially independent of glycaemic control [2,3]. Human studies further support a potential disease-modifying role, reporting improvements in nerve morphology and electrophysiological parameters [16,37]. Preservation of neural integrity may reduce repetitive, unnoticed trauma, thereby lowering the risk of ulcer formation and infection.

At the vascular level, GLP-1 receptor signalling improves endothelial function, enhances nitric oxide bioavailability, and mitigates diabetes-related microvascular dysfunction [13,18,19,38]. Although clinical data on distal microcirculation remain limited, mechanistic studies indicate reductions in oxidative stress and inflammatory activation within endothelial cells, potentially improving perfusion of the vasa nervorum and the microcirculatory network of the foot. Such effects are particularly relevant in neuro-ischaemic diabetic foot disease, where impaired blood supply compromises both nerve integrity and wound healing.

Emerging evidence also supports a direct role of GLP-1RAs in wound healing and tissue repair. Preclinical studies have demonstrated accelerated wound closure through enhanced angiogenesis, increased VEGF expression, modulation of macrophage polarisation towards a reparative phenotype, and stimulation of keratinocyte and fibroblast migration via PI3K/Akt- and ERK-dependent pathways [10,31,35,39]. These mechanisms directly address key pathological features of diabetic foot ulcers, including chronic inflammation, impaired angiogenesis and delayed re-epithelialisation. Recent systematic and narrative reviews suggest that these pleiotropic actions may translate into improved ulcer outcomes and reduced progression to severe infection or amputation [29,30].

**Figure 2:**
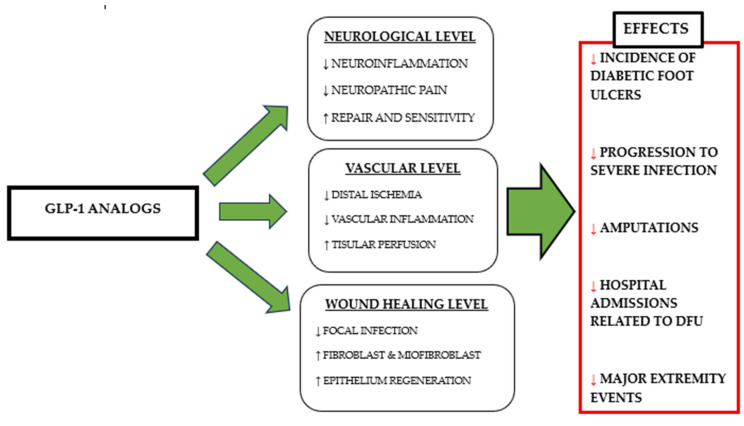
GLP-1 receptor agonists exert pleiotropic effects on peripheral nerves, microcirculation, and inflammation and tissue repair processes. The convergence of these neurological, vascular, and immune-repair mechanisms may explain the reduction in diabetic foot ulcers, amputations, and hospitalisations observed in recent clinical and population studies.

Pharmacokinetic properties also contribute to these effects. Molecules such as semaglutide exhibit prolonged half-lives and resistance to enzymatic degradation, allowing for the sustained activation of the GLP-1 receptor and reinforcing both metabolic stability and the consistency of extra-pancreatic actions. Taken together, these mechanisms position GLP-1RAs as therapeutic agents capable of influencing not only glycaemic and weight control, but also broader pathophysiological processes directly relevant to the progression and prognosis of diabetic foot disease (Figure 2).

### 4.3. Integration with Clinical Evidence and Limitations

Importantly, the mechanistic plausibility outlined above aligns with real-world clinical evidence. Large observational studies and cohort analyses report associations between GLP-1RA therapy and a reduced incidence of diabetic foot ulcers, lower rates of lower-limb amputation, and fewer limb-related adverse events compared with other glucose-lowering strategies [14,15,33]. Given that ulcer complications and amputations are major drivers of hospital admission in patients with diabetes, these findings provide a biologically credible explanation for the observed reduction in hospitalisation-related outcomes in population-based studies.

Nevertheless, the current evidence base remains predominantly observational, and causality cannot yet be firmly established. Prospective trials specifically designed to evaluate diabetic foot-related endpoints—including neuropathy progression, ulcer healing, and hospitalisation rates—are still lacking. Future studies integrating clinical outcomes with mechanistic biomarkers of neural and microvascular function will be essential to clarify the precise role of GLP-1RAs in the management of diabetic foot disease.

### 4.4. Limitations

This review has several limitations that should be acknowledged. First, owing to its narrative design, the selection and synthesis of evidence were qualitative rather than systematic, and no formal risk-of-bias assessment was performed. Second, most of the available clinical evidence regarding the effects of glucagon-like peptide-1 receptor agonists on diabetic foot-related outcomes derives from observational studies and real-world data, which precludes definitive conclusions regarding causality. Third, heterogeneity exists among studies in terms of patient populations, outcome definitions, and follow-up duration, limiting direct comparability. Finally, dedicated prospective trials specifically designed to evaluate diabetic foot-related endpoints remain scarce. These limitations highlight the need for well-designed future studies to confirm and extend the findings discussed.

## 5. Conclusions

Accumulating clinical and translational evidence suggests that glucagon-like peptide-1 receptor agonists may exert beneficial effects on several key components of diabetic foot disease, including peripheral neuropathy, ulcer progression, and severe lower-extremity outcomes that frequently lead to hospitalisation. Beyond their glucose-lowering properties, GLP-1 RAs display pleiotropic actions involving neuroprotective, vascular and anti-inflammatory mechanisms that are biologically relevant to the pathophysiology of diabetic foot disease.

Observational studies and real-world data consistently report associations between GLP-1 RAs therapy and a reduced incidence of diabetic foot ulcers, amputations, and limb-related adverse events when compared with other glucose-lowering strategies. These findings provide a plausible mechanistic and clinical framework supporting a potential role for GLP-1 RAs in modifying the course of diabetic foot disease and reducing its associated healthcare burden.

Nevertheless, current evidence remains largely observational, and causality cannot yet be firmly established. Prospective studies specifically designed to evaluate diabetic foot-related outcomes are required to confirm these observations and to better define the role of GLP-1 RAs in the multidisciplinary management of diabetic foot disease.

## Figures and Tables

**Table 1:** Role of peripheral neuropathy in the pathophysiology and clinical course of diabetic foot disease.

Sensory Neuropathy	Motor Neuropathy	Autonomic Neuropathy
Loss of protective sensation (pain, pressure, and temperature).	Intrinsic muscle atrophy and biomechanical imbalance of the foot.	Reduced sweating and impaired cutaneous vascular regulation.
Repeated unperceived microtrauma, development of painless ulcers, delayed diagnosis, and increased risk of infection.	Foot deformities (claw toes, bony prominences), increased plantar pressures, hyperkeratosis, and pressure-related ulceration.	Dry and fissured skin, impaired skin barrier function, increased susceptibility to infection, and poor wound healing.

**Table 2:** Primary clinical and observational studies evaluating the association between GLP-1 RAs and diabetic foot-related outcomes.

Author (Year)	Study Design/Data Source	Population	Exposure	Main Outcomes Assessed	Key Findings
Werkman et al. (2024) [14]	Population-based cohort study (CPRD Aurum database).	Patients with T2DM.	GLP-1 RAs vs. other glucose-lowering agents.	Diabetic foot ulcers, lower-limb amputations, and foot-related hospitalisations.	GLP-1 RAs were associated with a significantly lower risk of diabetic foot ulcers, amputations, and related hospitalisations compared with other antidiabetic treatments.
Hong et al. (2025) [15]	Nationwide retrospective cohort study.	>180,000 matched patients with T2DM.	GLP-1 RAs vs. SGLT2 inhibitors.	Major and minor lower-extremity amputations, mortality.	GLP-1 RAs were associated with a lower incidence of lower-limb amputations and reduced mortality compared with SGLT2 inhibitors.
Lewis et al. (2025) [26]	Real-world database analysis (TriNetX network).	Patients with active diabetic foot ulcers.	Semaglutide vs. non-GLP-1RA therapies.	Wound-healing complications, infections, and amputations.	Semaglutide use was associated with reduced rates of non-healing wounds, recurrent infections, and both minor and major amputations.
Caruso et al. (2025) [28]	Observational cohort study.	Patients with T2DM and peripheral arterial disease or foot ulcers.	Semaglutide vs. standard care.	Major adverse limb events, amputations.	Semaglutide was associated with a 23% reduction in major adverse limb events and a 50% reduction in amputations.
Lu and Guo (2023) [25]	Meta-analysis of observational studies (>2 million patients).	Patients with T2DM.	GLP-1 RAs vs. SGLT2 inhibitors and DPP-4 inhibitors.	Lower-limb amputation.	GLP-1 RAs showed a favourable safety profile, with no increased amputation risk and a trend toward lower risk versus other drug classes.

**Table 3:** Preclinical (animal) studies evaluating the effects of GLP-1 receptor agonists on mechanisms relevant to diabetic foot disease.

Study	Experimental Model	GLP-1 RA	Main Mechanistic Findings	Relevance to DFD
Gong et al., 2014 [31]	Streptozotocin-induced diabetic rats.	Liraglutide	Reduced oxidative stress, decreased inflammatory cytokine expression, and improved nerve conduction velocity.	Neuroprotection and attenuation of diabetic peripheral neuropathy.
Lu et al., 2023 [25]	Diabetic mouse model.	Exenatide	Suppressed microglial activation and neuronal apoptosis; improved nociceptive thresholds.	Preservation of peripheral nerve integrity.
Yang et al., 2019 [19]	Diabetic rodents with cutaneous wounds.	Liraglutide	Accelerated wound closure via enhanced angiogenesis and increased VEGF expression.	Improved wound healing in diabetic foot ulcers.
Zhang et al., 2017 [4]	High-fat diet/STZ-induced diabetes model.	Semaglutide	Reduced systemic inflammation and endothelial dysfunction; improved microvascular perfusion.	Potential improvement of neuro-ischaemic diabetic foot pathology.
Lee et al., 2024 [30]	Diabetic rat neuropathy model.	Dulaglutide	Reduced oxidative stress and neuronal degeneration; improved peripheral nerve morphology.	Disease-modifying effects on diabetic neuropathy.

**Table 4:** Clinical (human) studies evaluating GLP-1 receptor agonists and outcomes relevant to diabetic foot disease.

Study	Study Design	Population	GLP-1 Receptor Agonist	Main Findings
Werkman et al., 2024 [14]	Retrospective cohort (CPRD Aurum).	Patients with T2DM.	GLP-1 RAs (class).	Reduced risk of diabetic foot ulceration, amputations, and foot-related hospitalisations compared with other glucose-lowering therapies.
Hong et al., 2025 [15]	Population-based comparative cohort.	Patients with T2DM.	GLP-1 RAs vs. SGLT2 inhibitors.	Lower incidence of major and minor amputations and diabetic foot ulcers.
Caruso et al., 2025 [28]	Observational cohort.	Patients with peripheral arterial disease or active foot ulcers.	Semaglutide	Reduced major adverse limb events and lower amputation rates.
TriNetX analysis, 2023 [26]	Real-world database analysis.	Patients with active diabetic foot ulcers.	Semaglutide	Reduced rates of non-healing ulcers, recurrent infections, and amputations.

## Data Availability

No new data were created or analyzed in this study.

## Abbreviations

The following abbreviations are used in this manuscript:

AMPK	Multidisciplinary Digital Publishing Institute
AgRP	Directory of open access journals
GLP-1 ra	GLP-1 receptor agonist
CART	Cocaine- and amphetamine-regulated transcript
CV	Cardiovascular
CVOT	Cardiovascular outcomes trial
DFU	Diabetic foot ulcer
DM	Diabetes mellitus
DPP-4	Dipeptidyl peptidase-4
PAD	Peripheral arterial disease
ERK	Extracellular signal-regulated kinase
GLP-1	Glucagon-like peptide-1
HbA1c	Glycated haemoglobin
IL	Interleukin
MALE	Major adverse limb events
MACE	Major adverse cardiovascular events
NF-kB	Nuclear factor kappa B
NO	Nitric oxide
NPY	Neuropeptide Y
DFD	Diabetic foot disease
PI3K	Phosphoinositide 3-kinase
POMC	Pro-opiomelanocortin
ROS	Reactive oxygen species
SGLT2i	Sodium–glucose cotransporter-2 inhibitors
TNF-α	Tumour necrosis factor alpha
VEGF	Vascular endothelial growth factor
